# Supplementary material for: Yersinia pestis Actively Inhibits the Production of Extracellular Vesicles by Human Neutrophils
Source: J Extracell Vesicles. 2025 Apr 16;14(4):e70074. doi: 10.1002/jev2.70074 (PMC12003101; doi:10.1002/jev2.70074)
Supplement: Supplementary file 1 — Supporting Information [file JEV2-14-e70074-s001.docx]

**
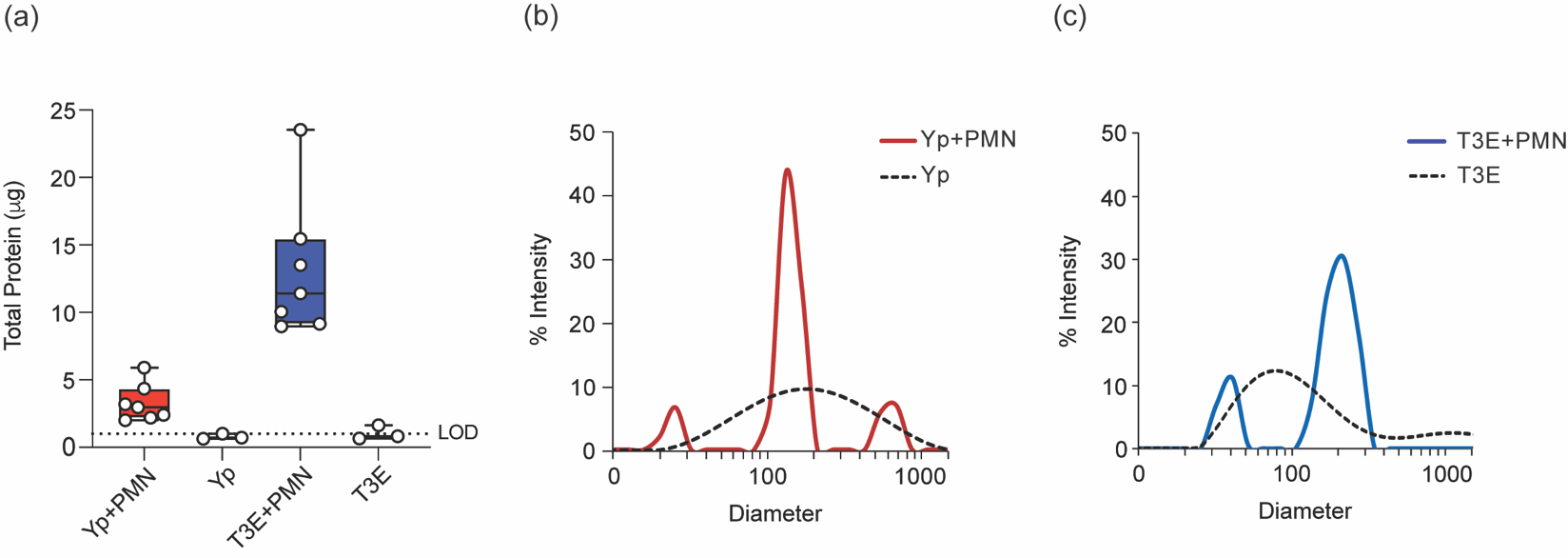
Supplemental Figure S1**: **Negligible OMV contamination within EV prep**

Equivalent amounts of WT *Y. pestis* (red) or T3E *Y. pestis* (blue) was incubated with or without hPMNs at an MOI of 50 for 1 h prior to EV isolation. (a) Protein quantification was performed using Protelite Fluorometric Protein Quantification Kit optimized for the Qubit fluorometer, limit of detection (LOD) = 1μg. Each point represents an independent biological replicate. (b-c) DLS analysis depicting EV profiles comparatively. Representative results of 3 independent experiments.

**
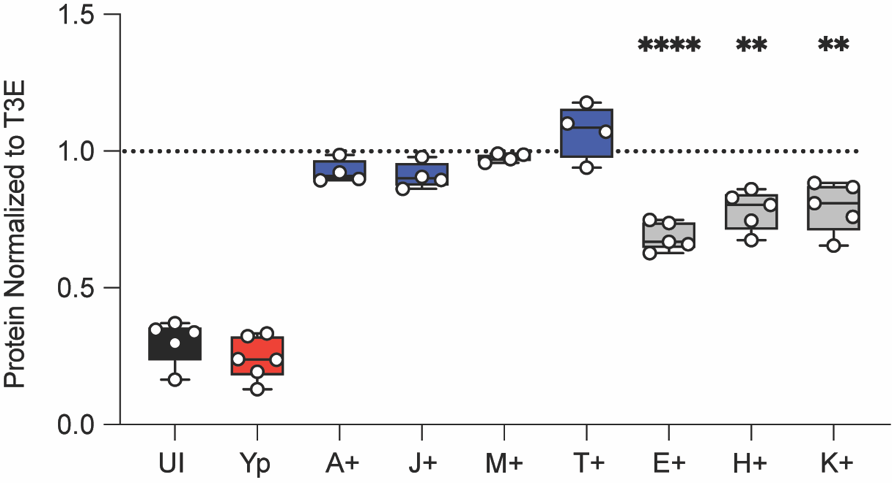
Supplemental Figure S2: Effect of individual Yop effectors on hPMN EVs.**

EVs were isolated from uninfected hPMNs (UI) or hPMNs that were infected with WT *Y. pestis* (Yp) or with a *Y. pestis* mutant expressing only YopA (A+), YopJ (J+), YopM (M+), YopT (T+), YopE (E+), YopH (H+), or YopK (K+). Total protein in each EV sample was quantified and normalized to the T3E control to account for donor variability. Recorded statistical significance is relative to T3E. One-way ANOVA with Dunnett’s multiple comparisons test to T3E with Geisser-Greenhouse correction; **= p≤0.005; ****=p≤0.0001 (n=4-6).


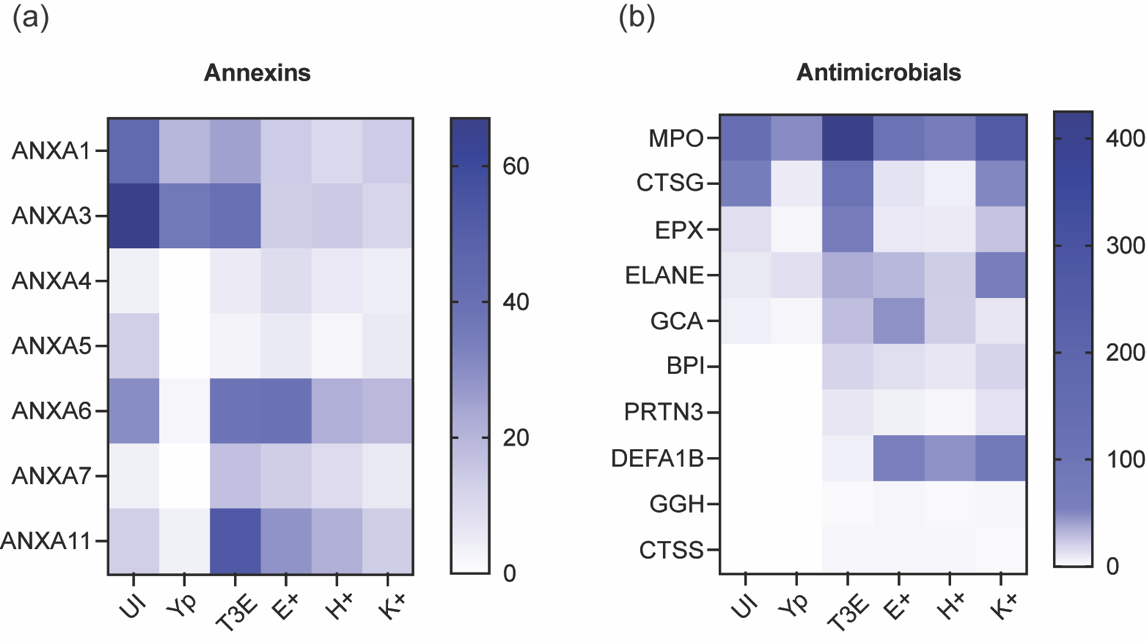


**Supplemental Figure S3: Impact of individual Yop effectors on EV protein packaging**

EVs elicited from hPMNs infected with *Y. pestis* mutants expressing a single Yop effector were analyzed via mass spectrometry. Comparative enrichment of annexin (a) and antimicrobial proteins (b).
